# Supplementary material for: Resolving the full spectrum of human genome variation using Linked-Reads
Source: Genome Res. 2019 Apr;29(4):635–45. doi: 10.1101/gr.234443.118 (PMC6442396; doi:10.1101/gr.234443.118)
Supplement: Supplemental Material [file supp_gr.234443.118_Supplemental_longranger-2.2.zip › longranger-2.2.tar.bz2!/lr2.2-public/lib/stan/cmdstan/stan/doc/api/html/agrad_2fwd_2matrix_2log__determinant_8hpp_source.html]

Stan: src/stan/agrad/fwd/matrix/log\_determinant.hpp Source File


|  |  |
| --- | --- |
|  | Stan  2.5.0  probability, sampling & optimization |


- Main Page
- Related Pages
- Namespaces
- Classes
- Files

- File List
- File Members

All Classes Namespaces Files Functions Variables Typedefs Enumerations Enumerator Friends Macros Pages

- src
- stan
- agrad
- fwd
- matrix

log\_determinant.hpp

Go to the documentation of this file.

1 #ifndef STAN\_\_AGRAD\_\_FWD\_\_MATRIX\_\_LOG\_DETERMINANT\_HPP

2 #define STAN\_\_AGRAD\_\_FWD\_\_MATRIX\_\_LOG\_DETERMINANT\_HPP

3

4 #include <vector>

5 #include <boost/math/tools/promotion.hpp>

6 #include <stan/math/matrix/Eigen.hpp>

7 #include <stan/agrad/fwd/fvar.hpp>

8 #include <stan/agrad/fwd/matrix/typedefs.hpp>

9 #include <stan/agrad/fwd/matrix/determinant.hpp>

10 #include <stan/agrad/fwd/functions/fabs.hpp>

11 #include <stan/agrad/fwd/functions/log.hpp>

12 #include <stan/math/error\_handling/matrix/check\_square.hpp>

13

14 namespace stan {

15  namespace agrad {

16

17  template<typename T, int R,int C>

18  inline

19  fvar<T>

20  log\_determinant(const Eigen::Matrix<fvar<T>, R, C>& m) {

21  stan::math::check\_square("log\_determinant(%1%)",m,"m",(double\*)0);

22

23  return stan::agrad::log(stan::agrad::fabs(stan::agrad::determinant(m)));

24  }

25  }

26 }

27 #endif

stan::agrad::fabs

fvar< T > fabs(const fvar< T > &x)

**Definition:** fabs.hpp:18

stan::agrad::fvar

**Definition:** fvar.hpp:13

log.hpp

typedefs.hpp

fvar.hpp

determinant.hpp

Eigen.hpp

stan::agrad::log

fvar< T > log(const fvar< T > &x)

**Definition:** log.hpp:15

stan::agrad::determinant

fvar< T > determinant(const Eigen::Matrix< fvar< T >, R, C > &m)

**Definition:** determinant.hpp:21

stan::agrad::log\_determinant

fvar< T > log\_determinant(const Eigen::Matrix< fvar< T >, R, C > &m)

**Definition:** log\_determinant.hpp:20

stan::math::check\_square

bool check\_square(const char \*function, const Eigen::Matrix< T\_y, Eigen::Dynamic, Eigen::Dynamic > &y, const char \*name, T\_result \*result)

Return true if the specified matrix is square.

**Definition:** check\_square.hpp:24

fabs.hpp

check\_square.hpp


---

[ Stan Home Page ]

*© 2011–2014,
Stan Development Team.*
